# Clinical usefulness of geriatric assessment in elderly patients with unresectable hepatocellular carcinoma receiving sorafenib or lenvatinib therapy

**DOI:** 10.1002/cnr2.1613

**Published:** 2022-03-18

**Authors:** Shuhei Sekiguchi, Kaoru Tsuchiya, Yutaka Yasui, Kento Inada, Sakura Kirino, Koji Yamashita, Yuka Hayakawa, Leona Osawa, Mayu Higuchi, Kenta Takaura, Chiaki Maeyashiki, Shun Kaneko, Nobuharu Tamaki, Hiroyuki Nakanishi, Jun Itakura, Yuka Takahashi, Yasuhiro Asahina, Ryuichi Okamoto, Masayuki Kurosaki, Namiki Izumi

**Affiliations:** ^1^ Department of Gastroenterology and Hepatology Musashino Red Cross Hospital Musashino‐shi Japan; ^2^ Department of Gastroenterology and Hepatology Tokyo Medical Dental University Bunkyo‐Ku Japan; ^3^ NAFLD Research Center, Department of Medicine University of California San Diego La Jolla California USA

**Keywords:** 1, Geriatric 8 score, hepatocellular carcinoma 2, lenvatinib 4, sorafenib 3

## Abstract

**Background:**

Therapeutic strategies for unresectable hepatocellular carcinoma (u‐HCC) in geriatric patients are important for real‐world practice. However, there remain no established biomarkers or therapeutic strategies regarding the best second‐line agent after atezolizumab plus bevacizumab therapy.

**Aim:**

In this study, we investigated the usefulness of modified Geriatric 8 (mG8) score in examining elderly patients (≥75 years old) with unresectable hepatocellular carcinoma (u‐HCC) using sorafenib or lenvatinib as first‐line therapy.

**Methods and results:**

This study assessed 101 elderly patients with u‐HCC for their mG8 score (excluding elements of age from 8 items) and classified them into 2 groups according to their mG8 score: ≥11 as the high‐score group and ≤ 10 as the low‐score group. Among those taking sorafenib, no significant differences were noted in overall survival (OS) and progression free survival (PFS) between low and high mG8 score groups. Only modified albumin–bilirubin (ALBI) grade (2b/3 vs. 1/2a: HR 0.34; 95% CI, 0.17–0.69; *p* = .0029) was significantly associated with OS. Among those taking lenvatinib, patients with a high mG8 score (*n* = 26) had longer survival than those with a low mG8 score (*n* = 10) (20.0 months vs. 7.7 months: HR 0.31, 95% CI 0.11–0.89; *p* = .029). Intrahepatic tumor volume (<50% vs. ≥50%: HR 16.7; 95% CI, 1.71–163; *p* = .016) and α‐fetoprotein (AFP) (<400 vs. ≥400: HR 3.38; 95% CI 0.84–19.7; *p* = .031) remained significant factors independently associated with OS.

**Conclusions:**

The mG8 score may contribute to making a decision when considering either sorafenib or lenvatinib as a treatment option for u‐HCC in elderly patients.

## 1 BACKGROUND

Hepatocellular carcinoma (HCC) has been identified as the most common cancer in the liver and one of the leading causes of cancer‐related deaths worldwide.^1^ HCC affects a heterogeneous population, but recently, the number of elderly cases has been increasing. Thus, therapeutic strategies for unresectable hepatocellular carcinoma (u‐HCC) in geriatric patients are important for real‐world practice. The International Society of Geriatric Oncology published a recommendation that elderly cancer patients should be evaluated via a geriatric assessment (GA). Included in the GA is the Geriatric 8 (G8)^2^ screening tool consisting of eight items dealing with age, food intake, weight loss, mobility, neuropsychological problems, body mass index, prescription drugs, and a self‐perception of health using Mini‐Nutritional Assessment questionnaire. The G8 score ranges from 0 to 17, with a higher score suggestive of a better health status. G8 has already shown prognostic value regarding survival outcomes in elderly patients with various cancers and hematological malignancies.^3^, ^4^, ^5^, ^6^

For the treatment of u‐HCC, tyrosine kinase inhibitors (TKIs) such as sorafenib,^7^, ^8^ and regorafenib,^9^ lenvatinib,^10^ and cabozantinib^11^ have been approved in many countries. Ramucirumab,^12^ a vascular endothelial growth factor‐2 (VEGFR‐2) antibody, has also been administered in u‐HCC patients with high AFP levels (≥400 ng/mL) as second‐line treatment. Furthermore, combination therapy of atezolizumab plus bevacizumab^13^ was approved in the United States and Japan in 2020 and has since become the first‐line treatment for u‐HCC. However, there remain no established biomarkers or therapeutic strategies regarding the best second‐line agent after atezolizumab plus bevacizumab therapy. In most cases, sorafenib or lenvatinib is chosen because these were the previous first‐line agents before combination therapy was recommended. Thus, in this study, we investigated the usefulness of mG8 in elderly u‐HCC patients who received sorafenib or lenvatinib as first‐line treatment and proposed a therapeutic strategy for such patients treated with TKI.

## 2 MATERIALS AND METHODS

### 2.1 Patients

From January 2014 to February 2021, 189 patients with u‐HCC received sorafenib or lenvatinib as first‐line therapy at Musashino Red Cross Hospital. Among these, a total of 101 elderly patients (aged ≥ 75 years) were included. The Japan Gerontological Society proposed the age classification in 2017.^14^ The proposal included the definition that aged from 65 to 74 years was pre‐old age and aged over 75 years was old age. According to the proposal, we introduced a cutoff age of 75 years in this study. The diagnosis of HCC was based on the guidelines proposed by either the Liver Cancer Study Group of Japan,^15^ the European Association for the Study of the Liver,^16^ or the American Association for the Study of Liver Diseases.^17^ The treatment for HCC was discussed by a tumor board. The study was conducted according to the guide‐lines of the Declaration of Helsinki and approved by the Institutional Review Board (or Ethics Committee) of Musashino Red Cross Hospital (protocol code: 1099, June 17, 2020). Informed consent was obtained from all subjects involved in the study.

### 2.2 Treatment protocol

Sorafenib was administered from July 2009 until March 2018, and after April 2018, when lenvatinib became available, the first‐line agent was decided after discussing with the attending physician. As regards the standard doses of chemotherapy, sorafenib was given at 800 mg/day, while lenvatinib was given at 12 and 8 mg/day for patients ≥60 and <60 kg, respectively. We then reduced the drug's initial dose considering patients’ condition and modified this according to the presence of vascular endothelial growth factor‐2 (VEGFR‐2). Antitumor responses were determined according to the modified Response Evaluation Criteria in Solid Tumor (mRESIST), using dynamic computed tomography (CT) or magnetic resonance imaging (MRI) within 4–8 weeks and every 8 weeks thereafter. AEs were then graded at every visit based on Common Terminology Criteria for Adverse Events(CTCAE) version 5.0. Informed consent was obtained from all subjects involved in the study.

### 2.3 Evaluation of geriatric patients with the functional score

The G8 score^2^ is normally used to assess elderly people, specifically regarding their functional decline, in the form of a multi‐item test. The G8 screening tool consists of eight items as follows: food intake, weight loss, mobility, neuropsychological problems, body mass index, medications, self‐impression about health, and age. In this study, we used a modified G8 (mG8) score, which consists of seven items dealing with food intake, weight loss, mobility, neuropsychological problem, body mass index, prescription drugs, and self‐perception of health. Age was excluded from our mG8 score, because age alone does not reflect functional status in the aging process. In this group, the median mG8 score was 11, so a score of ≥11 was defined as a high score, while ≤10 was a low score.

### 2.4 Statistical analysis

Statistical analyses were then performed using the EZR version 2.23 (Saitama Medical Centre, Jichi Medical University, Shimotsuke, Japan).^18^ The primary endpoint was the duration of overall survival (OS). OS was measured as the time from starting treatment with sorafenib or lenvatinib until the date of death from any cause or censored at the last follow‐up date. OS was analyzed using the Kaplan–Meier method via the log‐rank test and Cox proportional hazards regression analysis. Outcome variables were analyzed using the *χ* ^2^ test or Fisher's exact test for categorical variables, while the Mann–Whitney U test was used for continuous variables. A Cox proportional regression hazard model was used to examine predictors of OS. A *p*‐value of <.05 was considered statistically significant.

## 3 RESULTS

### 3.1 Baseline patient characteristics

In total, 101 patients were enrolled in this study. The baseline characteristics of these patients were shown in Table 1; these were similar between sorafenib and lenvatinib patients, except for extrahepatic spread. There was no difference in the mG8 score between the two groups. The baseline characteristics according to mG8 score in each therapy group are shown in Tables S1 and S2.

**TABLE 1:** Baseline characteristics

Factor	Group	Sorafenib (*n* = 65)	Lenvatinib (*n* = 36)	*p*‐Value
BCLC stage, *n* (%)	A	1 (1.5)	0 (0.0)	0.165
	B	32 (49.2)	24 (66.7)	
	C	32 (49.2)	12 (33.3)	
	0	44 (67.7)	21 (58.3)	0.21
ECOG‐PS, *n* (%)	1	18 (27.7)	15 (41.7)	
	2	3 (4.6)	0 (0.0)	
Gender, *n* (%)	F	20 (30.8)	9 (25.0)	0.648
	M	45 (69.2)	27 (75.0)	
Age, years median (range)		80 (75, 98)	81 (75, 93)	0.217
Etiology, *n* (%)	ALD	8 (12.3)	8 (22.2)	0.487
	HBV	3 (4.6)	1 (2.8)	
	HBV + HCV	1 (1.5)	0 (0.0)	
	HCV	38 (58.5)	16 (44.4)	
	Others	15 (23.1)	11 (30.6)	
Major vascular invasion (%)	No	56 (86.2)	32 (88.9)	0.767
	Yes	9 (13.8)	4 (11.1)	
Extrahepatic spread (%)	Yes	27 (41.5)	7 (19.4)	0.029
	No	38 (58.5)	29 (80.6)	
Tumor volume ≥ 50% (%)	No	57 (89.1)	33 (91.7)	1
	Yes	7 (10.9)	3 (8.3)	
Child‐Pugh score (%)	5	30 (46.2)	21 (60.0)	0.464
	6	27 (41.5)	10 (28.6)	
	7	7 (10.8)	3 (8.6)	
	8	1 (1.5)	1 (2.9)	
ALBI score, median (range)		−2.14 (−2.93, −1.33)	−2.36 (−3.21, −1.35)	0.105
Baseline DCP, median (range)		267 (8.90, 54 104)	606 (10.2, 427 125)	0.442
Baseline AFP median (range)		36.6 (1.80, 69 100)	30.4 (1.6, 97 455)	0.633
mG8 score		11 (8, 15)	12 (8, 14)	0.09

Abbreviations: BCLS stage: Barcelona Clinic Liver Cancer stage; ECOG‐PS: the Eastern Cooperative Oncology Group Scale of Performance Status; ALBI score: albumin–bilirubin score; DCP: des‐gamma‐carboxy prothrombin; AFP: α‐fetoprotein;mG8 score: modified Geriatric 8 score.

**FIGURE 1:**
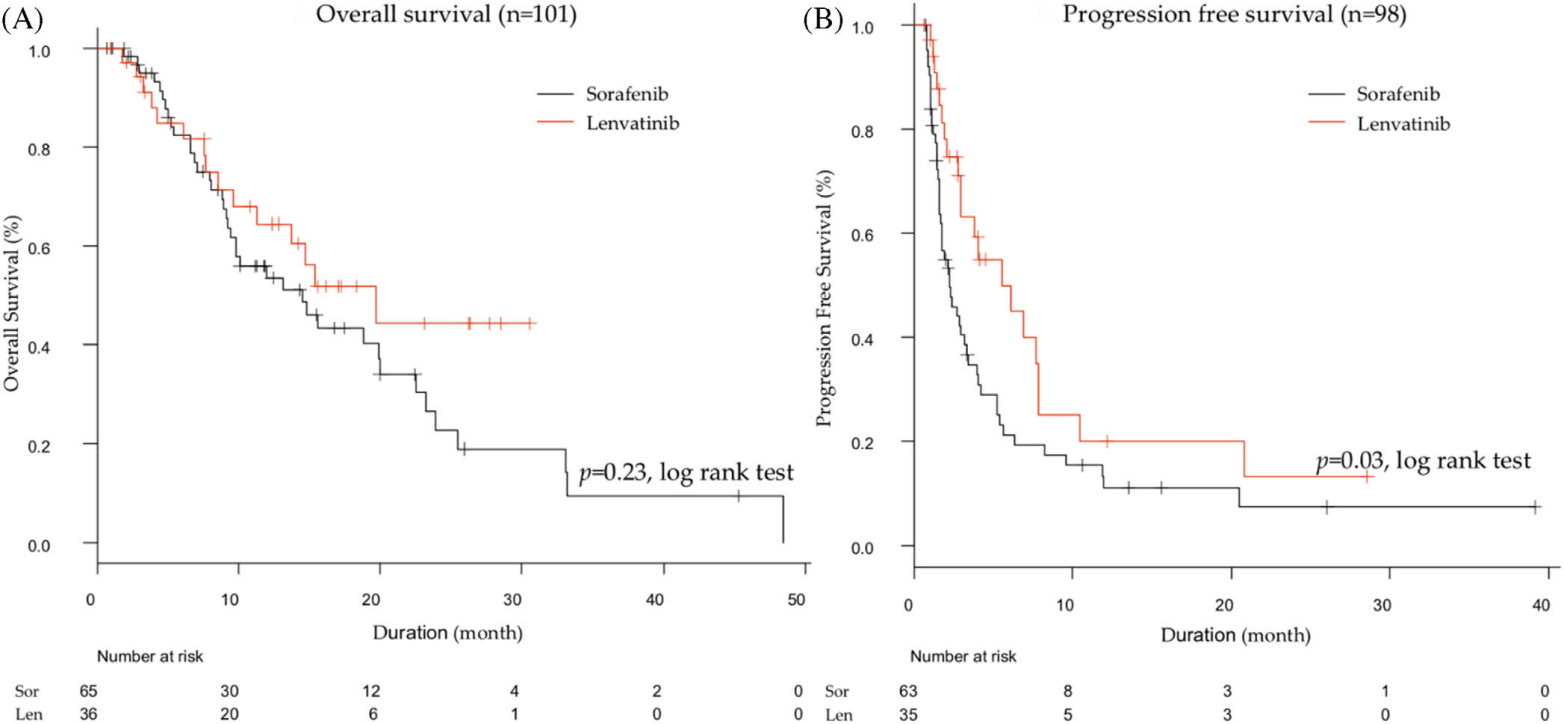
Comparison between sorafenib and lenvatinib patients in overall (A) and progression‐free survival (B)

The median OS and PFS in all patients were 15.0 and 3.0 months, respectively. The median treatment duration of sorafenib and lenvatinib were 2.5 and 3.5 months, respectively. The median OS was 14.7 months (95% CI, 9.4–20.3) in the sorafenib group, while this was 20.0 months (95% CI, 9.8 to not reached) in the lenvatinib group, but this was not significantly different between the two (HR 0.69, 95% CI 0.38–1.26; *p* = .23) (Figure 1A). However, a significant difference was noted in PFS between the two groups (5.6 vs. 2.3 months, HR 0.57, 95% CI 0.34–0.95, *p* = .03) (Figure 1B). In the sorafenib group, the objective response rate (ORR) was 12.7%, and the disease control rate (DCR) was 65.5%. On the other hand, in the lenvatinib group, the ORR was 56.5%, while the DCR was 73.9%. The ORR was significantly better in the lenvatinib group (56.5% vs. 12.7%, *p* < .001), but there was no significant difference in DCR between the two groups (73.9% vs. 65.5%, *p* = .80).

### 3.2 AEs during the therapy

The AEs noted during sorafenib and lenvatinib treatment are shown in Table 2. The major AEs (≥40%) were hand‐foot skin reactions (HFSRs) (44.6%) and hypertension (43.1%) in sorafenib group, while these were hypertension (63.9%), general fatigue (58.3%), and appetite loss (52.8%) in the lenvatinib group. The discontinuation rate related to AEs was 30.8% for the sorafenib group and 44.4% for the lenvatinib group. We further classified sorafenib and lenvatinib into two groups according to their mG8 score, that is, the low‐score (mG8 ≤ 10) group and the high‐score (mG8 ≥ 11) group. In both sorafenib and lenvatnib groups, the total number of HFSRs was higher among patients with high mG8 scores (*p* = .003 and *p* = .016, respectively).

**TABLE 2:** Adverse events

		Sorafenib			Lenvatinib		
		Total	mG8 low	mG8 high	Total	mG8 low	mG8 high
Adverse events		*n* = 65	*n* = 22	*n* = 43	*n* = 36	*n* = 10	*n* = 26
Hypertension, *n* (%)	Any grade	28 (43.1)	7 (31.8)	21 (48.8)	23 (63.9)	7 (70.0)	16 (61.5)
	Grades 3–4	1 (1.5)	0 (0)	1 (2.3)	3 (8.3)	0 (0)	3 (11.5)
Hand‐foot skin reactions, *n* (%)	Any grade	29 (44.6)	4 (18.2)^a^	25 (58.1)^a^	11 (30.6)	0 (0)^b^	11 (42.3)^b^
	Grades 3–4	3 (4.6)	0 (0)	3 (7)	0 (0)	0 (0)	0(0)
Diarrhea, *n* (%)	Any grade	12 (18.5)	4 (18.2)	8 (18.6)	9 (25.0)	2 (20.0)	7 (26.9)
	Grades 3–4	3 (4.6)	2 (9.1)	1 (2.3)	2 (5.6)	1 (10.0)	1 (3.8)
Liver dysfunction, *n* (%)	Any grade	10 (15.4)	3 (13.6)	7 (16.3)	8 (22.2)	4 (40.0)	4 (15.4)
	Grades 3–4	2 (3.1)	1 (4.5)	1 (2.3)	1 (2.8)	0 (0)	1 (3.8)
Appetite loss, *n* (%)	Any grade	23 (35.4)	5 (22.7)	18 (41.9)	19 (52.8)	6 (60.0)	13 (50.0)
	Grades 3–4	2 (3.1)	1 (4.5)	1 (2.3)	1 (2.8)	1(10.0)	0 (0)
Fatigue, *n* (%)	Any grade	22 (33.8)	10 (45.5)	12 (27.9)	21 (58.3)	7 (70)	14 (53.8)
	Grades 3–4	1 (1.5)	0 (0)	1 (2.3)	0(0)	1 (3.58)	0 (0)

^a^ The total number of hand‐foot skin reactions (HFSRs) was higher among patients in the high mG8 group (*p* = .003).

^b^ The total number of hand‐foot skin reactions (HFSRs) was higher among patients in the high mG8 group (*p* = .016).

### 3.3 The impact of modified G8 score in sorafenib and lenvatinib therapy

**FIGURE 2:**
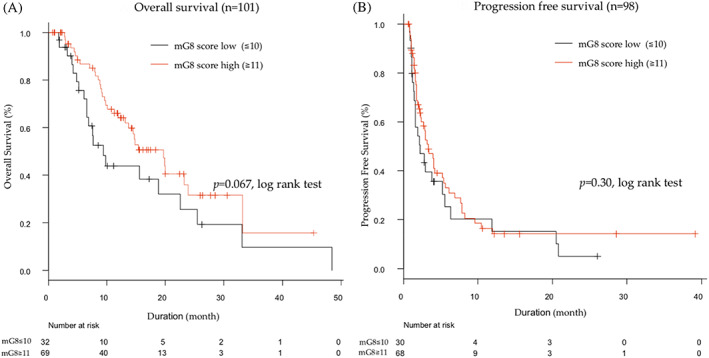
Overall survival and progression‐free survival outcomes in all patients. (A) Kaplan–Meier estimates of overall survival by mG8 scores. (B) Kaplan–Meier estimates of progression‐free survival by mG8 scores

**FIGURE 3:**
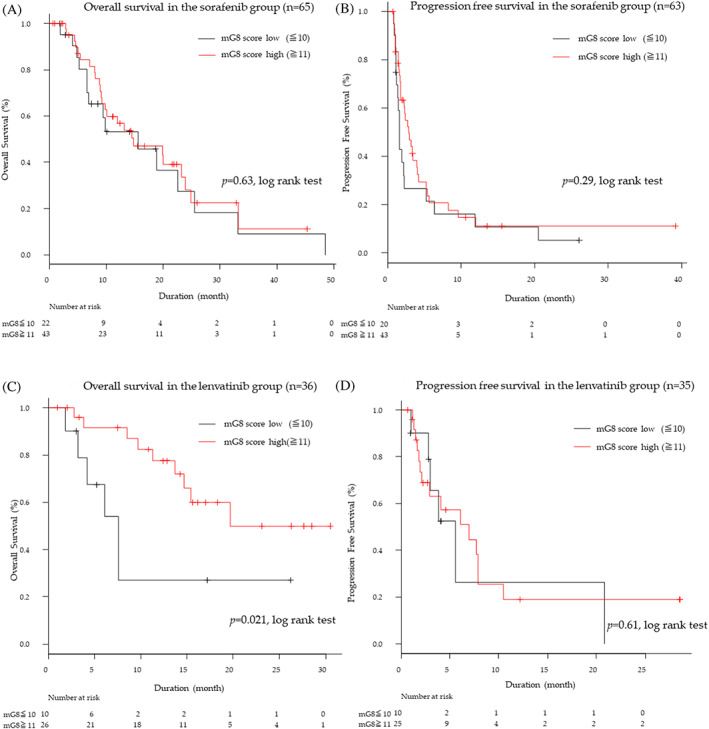
Overall survival and progression‐free survival outcomes in the sorafenib and lenvatinib group. (A) Kaplan–Meier estimates of overall survival in the sorafenib by mG8 scores. (B) Kaplan–Meier estimates of progression‐free survival in the sorafenib by mG8 scores. (C) Kaplan–Meier estimates of overall survival in the lenvatinib by mG8 scores. (D) Kaplan–Meier estimates of progression‐free survival in the lenvatinib by mG8 scores

We classified all patients into two groups based on their mG8 scores: the low‐score (mG8 ≤ 10) group (*n* = 32) and the high‐score (mG8 ≥ 11) group (*n* = 69). There were no significant differences in OS and PFS between low‐ and high‐score groups (Figure 2A,B). We further analyzed OS and PFS according to the mG8 score in each therapy separately. In the sorafenib group, the median OS and PFS were 15.8 and 1.6 months in the low‐score group, respectively, while in the high‐score group, these were 15.0 and 2.9 months, respectively (Figure 3A,B). There were no significant differences in OS and PFS between high‐ and low‐score groups. In the lenvatinib group, patients with high mG8 scores (*n* = 26) had longer survival than those patients with a low mG8 score (*n* = 10) (20.0 months vs. 7.7 months, HR 0.31, 95% CI 0.11–0.89; *p* = .029) (Figure 3C). However, there were no significant differences in PFS between low‐ and high‐score groups (6.2 months vs. 5.6 months, *p* = .61) (Figure 3D).

We classified mG8 high score (mG8 ≥ 11) patients into two groups based on their therapeutic agents: sorafenib group (*n* = 43) and lenvatinib group (*n* = 26). There were no significant differences in OS and PFS between sorafenib and lenvatinib groups (Figure S1A,B). We further classified mG8 low score (mG8 ≤ 10) patients into two groups based on their therapeutic agents: sorafenib group (*n* = 22) and lenvatinib group (*n* = 10). There were no significant differences in OS and PFS between sorafenib and lenvatinib groups (Figure S1A,B).

### 3.4 Factors associated with PFS

In the univariate analysis of the sorafenib group, only the modified ALBI grade (2b/3 vs. 1/2a: HR 0.48; 95% CI 0.27–0.88; *p* = .017) was associated with PFS. Among the patients treated with lenvatinib, in the univariate analysis, intrahepatic tumor volume (<50% vs. ≥50%: HR 44.1; 95% CI 3.77–515; *p* = .0025), and AFP(<400 vs. ≥400: HR 2.95; 95% CI 1.16–7.51; *p* = .023) were associated with PFS. In the multivariate analysis, intrahepatic tumor volume (<50% vs. ≥50%: HR 24.8; 95% CI 2.04–301; *p* = .012) was only a significant factor (Table S3).

### 3.5 Factors associated with OS

In the univariate analysis of all patients, modified albumin–bilirubin (ALBI) grade^19^, ^20^ (2b/3 vs. 1/2a: HR 0.45; 95% CI, 0.26–0.78; *p* = .0047), intrahepatic tumor volume (<50% vs. ≥50%: HR 3.31; 95% CI 1.47–7.45; *p* = .0038), DCP (<400 vs. ≥400: HR 2.23; 95% CI 1.28–3.89; *p* = .0047), and AFP (<400 vs. ≥400: HR 1.90; 95% CI 1.02–3.53; *p* = .042) were all associated with OS. In the multivariate analysis, modified ALBI grade (2b/3 vs. 1/2a: HR 0.38; 95% CI 0.21–0.68; *p* = .0011), intrahepatic tumor volume (<50% vs. ≥50%: HR 2.80; 95% CI 1.16–6.76; *p* = 0.022), and pretreatment DCP (<400 vs. ≥400: HR 2.08; 95% CI 1.15–3.75; *p* = 0.016) remained independent prognostic factors for OS (Table 3).

**TABLE 3:** Factors associated with OS

	All patients				sorafenib				lenvatinib			
	Univariate		Multivariate		Univariate		Multivariate		Univariate		Multivariate	
Factor	HR(95% CI)	*p*‐Value	HR(95% CI)	*p*‐Value	HR(95% CI)	*p*‐Value	HR(95% CI)	*p*‐Value	HR(95% CI)	*p*‐Value	HR(95% CI)	*p*‐Value
Gender												
Female	1				1				1			
Male	1.25(0.68–2.28)	0.47			1.24(0.6–2.42)	0.53			1.32(0.37–4.74)	0.67		
Age												
75–79	1				1				1			
≥80	1.26(0.72–2.2)	0.43			1.22(0.64–2.33)	0.55			1.11(0.38–3.27)	0.84		
ECOG‐PS												
1/2	1				1				1			
0	1.2(0.68–2.11)	0.54			0.98(0.49–1.97)	0.96			1.36(0.49–3.78)	0.56		
Major vascular invasion												
VP0/1/2	1				1				1			
VP3/4	1.88(0.83–4.22)	0.13			2.01(0.77–5.28)	0.16			1.81(0.4–8.18)	0.44		
Extrahepatic spread												
Yes	1				1				1			
No	0.97(0.55–1.71)	0.9			1.03(0.54–1.98)	0.92			1.16(0.33–4.12)	0.82		
Modified ALBI grade												
2b/3	1		1		1				1			
1/2a	0.45(0.26–0.78)	0.0047	0.38(0.21–0.68)	0.0011	0.34(0.17–0.69)	0.0029			0.78(0.25–2.46)	0.67		
mG8 score												
≤10	1				1				1			
≥11	0.60(0.34–1.05)	0.07			0.85(0.44–1.64)	0.63			0.31(0.11–0.89)	0.029	0.42(0.13–1.38)	0.15
Intrahepatic tumor volume												
<50%	1		1		1				1			
≥50%	3.31(1.47–7.45)	0.0038	2.80(1.16–6.76)	0.022	2.44(0.94–6.35)	0.067			13.6(2.19–84.3)	0.0051	16.7(1.71–163)	0.016
DCP												
<400 mAU/mL	1		1		1				1			
≥400 mAU/mL	2.23(1.28–3.89)	0.0047	2.08(1.15–3.75)	0.016	1.64(0.87–3.1)	0.13			4.99(1.36–18.3)	0.015	3.92(0.96–16)	0.057
AFP												
<400 mAU/mL	1		1		1				1		1	
≥400 mAU/mL	1.90(1.02–3.53)	0.042	1.12(0.58–2.19)	0.73	1.21(0.57–2.55)	0.63			4.61(1.37–15.5)	0.013	3.38(0.84–19.7)	0.031
TKI												
Sorafenib	1											
Lenvatinib	0.69(0.38–1.26)	0.23										

Abbreviations: ECOG‐PS: the Eastern Cooperative Oncology Group Scale of Performance Status; modified ALBI score: modified albumin–bilirubin score; mG8 score: modified Geriatric 8 score,DCP: des‐gamma‐carboxy prothrombin; AFP: α‐fetoprotein; TKI: tyrosine kinase inhibitor.

In the univariate analysis of the sorafenib group, only the modified ALBI grade (2b/3 vs. 1/2a: HR 0.34; 95% CI 0.17–0.69; *p* = .0029) was associated with OS, whereas there were no significant differences regarding gender, ECOG‐PS, age, major portal invasion, extrahepatic spread, extrahepatic tumor volume, AFP level, DCP level, and mG8 score (Table 3).

Among the patients treated with lenvatinib, in the univariate analysis, modified G8 score (≤10 vs. ≥11: HR0.31; 95% CI 0.11–0.89; *p* = .029), intrahepatic tumor volume (<50% vs. ≥50%: HR 13.6; 95% CI, 2.19–84.3; *p* = .0051), DCP (<400 vs. ≥400: HR 4.99; 95% CI 1.36–18.3; *p* = .015), and AFP(<400 vs. ≥400: HR 4.61; 95% CI 1.37–15.5; *p* = .013) were associated with OS. In the multivariate analysis, intrahepatic tumor volume (<50% vs. ≥50%: HR 16.7; 95% CI 1.71–163; *p* = 0.016), and AFP (<400 vs. ≥400: HR 3.38; 95% CI 0.84–19.7; *p* = 0.031) remained independent prognostic factors for OS (Table 3). In lenvatinib patients, patients with a high mG8 score survived significantly longer than patients with a low mG8 score. In lenvatinib group, there was no significant difference in the duration of medication, discontinuation rate due to AEs, and relative dose intensity during the initial 4 weeks of therapy, but transition rate to post‐treatment was higher in mG8 high‐score group than low‐score groups (Table 4).

**TABLE 4:** Factors for treatment in lenvanitib group

Factor	Low mG8 (*n* = 10)	High mG8 (*n* = 26)	*p*‐Value
Transition rate to posttreatment, *n* (%)	4 (40.0)	18 (78.3)	0.049
Discontinuation rate due to AEs, *n* (%)	6 (60.0)	10 (38.5)	0.285
4W‐RDI, median (range)	0.49 (0.07, 1.00)	0.79 (0.02, 1.00)	0.119
Duration of medication, median (range)	93.50 (4.00, 643)	106.00 (1.00, 868)	0.48

Abbreviation: 4W‐RDI: relative dose intensity during the initial 4 weeks of therapy.

## 4 DISCUSSION

To the best of our knowledge, this is the first study to show the usefulness of GA in elderly patients with u‐HCC treated with systemic sorafenib or lenvatinib as first‐line therapy. The median age of HCC patients has been rising because the number of patients with nonalcoholic steatohepatitis (NASH) has been increasing in many countries,^21^, ^22^ and such patients develop HCC at older ages compared to those with hepatitis B or C infection. The individual variations among these elderly patients were associated with the difficulty of deciding the most recommended therapy, because judging performance status is often more complicated than in nonelderly patients.

The usefulness of the G8 score in patients with cancer has already been reported in previous studies. Agemi et al.^23^ reported that an impaired G8 score (≤14) was an independent prognostic factor for OS in lung cancer patients. Yamada et al.^24^ demonstrated that a lower G8 score (<9.5) was associated with poorer self‐reliance rates in the 438 patients who had oral squamous cell carcinoma and were aged 75 years and older. In this study, we used the mG8 score, which consisted of seven variables, eliminating the effect of age, because in real‐world practice, there are super‐agers who maintain physical and mental ability in their 80s to 90s. Martinez‐Tapia et al.^25^ already proposed a different mG8 score consisting of six items, including weight loss, cognition/mood, performance status, self‐rated health status, polypharmacy, and history of heart failure/coronary heart disease. Their mG8 score also did not include age. Recently, Kaibori, et al^26^ reported perioperative maintenance of G8 score was an independent prognostic indicator for both RFS and OS in elderly HCC patients who received hepatectomy.

Our study revealed that in all patients, there was no significant difference in OS between groups with high and low mG8 scores. However, in the lenvatinib group, patients with low mG8 scores (≤10) had poorer OS than those with high mG8 scores (≥11). In the lenvatinib group, there was no significant difference in the duration of medication, discontinuation rate due to AEs, and relative dose intensity, but transition rate to posttreatment was higher in the high‐score group than in the low‐score group. Interestingly, our mG8 score was not a significant factor associated with OS in patients treated with sorafenib.

One of the speculations for the reason is inhibition of the fibroblast growth factor 19 (FGF19). It was already reported that FGF19 increased skeletal muscle mass and strength.^27^ Lenvatinib inhibits fibroblast growth factor (FGF) receptors 1–4, and FGF19 is a tumor biomarker of lenvatinib‐susceptible HCC.^28^ Using lenvatinib in patients with a high mG8 score would worsen general conditions, including muscle mass and strength, and may be associated with shorter OS.

In this study, the OS did not differ between patients treated with lenvatinib and sorafenib, although the PFS was significantly better in lenvaintib group than the sorafenib group. Similar results were already reported in the Phase 3 study of lenvatinib (REFLECT study)^10^ and also in the study in which the OS and PFS were compared between sorafenib and lenvatinib after first‐line atezolizumab plus bevacizumab in advanced HCC patient.^29^ There is still no understandable reason for the discrepancy, and prospective large cohort studies have been required.

In the treatment of advanced HCC, atezolizumab plus bevacizumab has become the first‐line therapy. However, there are no established biomarkers used as a basis for choosing the second‐line agent. According to a Phase 3 trial (REFLECT trial),^10^ lenvatinib showed noninferiority in OS compared to sorafenib. Our mG8 score can aid in determining a recommended therapeutic strategy in elderly patients with u‐HCC.

The cut‐off value of AFP was defined as 400 ng/mL in most recent studies for advanced HCC, according to the REACH^30^ and REACH‐2 study,^12^ while there was no established cut‐off value of DCP in patients with unresectable HCC. In this study, we adapted the cut‐off value of DCP as 400 mAU/mL according to the previous studies, which had revealed the association between DCP 400 mAU/ml and prognosis after living‐donor liver transplantation^31^ or liver resection.^32^ These studies also showed the relationship between DCP 400mAU/ml and biological malignant potential including microvascular invasion and poorly differentiated HCC.

There are certain limitations to our study. The study was a retrospective, single‐center study, and the number of patients was small. All therapeutic decisions, including the initial dose, dose modification, interruption duration, and post‐sorafenib or lenvatinib therapies, were made by each individual investigator. Moreover, our modified G8 score was not validated in any other cohort, and the subgroup analysis should be carefully evaluated because of a minimal number.

In advanced HCC era, immuno‐combination therapy is recommended as the first line. Most advanced HCC patients were a candidate for sequential therapy with multiple molecular targeted agents. Modified G8 score would be a valuable tool to make a therapeutic strategy for the sequence. It was already reported that 64% of the geriatric patients took more than four kinds of drugs in Tokyo, Japan, while 32.3% of the 80–89 years patients was prescribed within 1–4 drug.^33^ The G8 score was proposed in 2012, and the diversity in geriatric patients has been promoted. Even though our study had a small number of patients, the mG8 score would be meaningful in real‐world practice, especially in a super‐aging society. It is the first study about modified G8 score, and we used the median value of mG8 score as a cut‐off value. It is necessary to evaluate the cut‐off value of mG8 in a large cohort study.

EASL recommended considering the several factors, including patient characteristics and comorbidities, when we selected a second‐line agent.^34^ We think the mG8 score is one of the patient characteristics, which may contribute to making a therapeutic strategy in geriatric patients with unresectable HCC. Further studies with a large cohort should be conducted to provide the clinical value of mG8 score. This study was the first report to reveal the usefulness of the GA in elderly patients with unresectable HCC who received systemic therapy. These results can help inform therapeutic strategies based on an individual patient's status. Further investigation in a larger cohort is thus necessary to establish the recommended therapies in each patient. Our results suggest that the grouping according to mG8 score is useful for selecting a TKI for elderly patients with HCC. In the manuscript about updated BCLC staging system (BCLC 2022), a multiparametric evaluation at multidisciplinary tumor boards is recommended,^35^ and a modified G8 score would become a useful parameter, especially in aged or super‐aged societies.

## 5 CONCLUSIONS

The mG8 score may contribute to making a decision when considering either sorafenib or lenvatinib as a treatment option for u‐HCC in elderly patients.

## AUTHOR CONTRIBUTIONS

**Kaoru Tsuchiya:** Conceptualization (equal); data curation (equal); methodology (equal); project administration (equal); resources (equal); writing – original draft (equal). **Yutaka Yasui:** Resources (equal). **Kento Inada:** Resources (equal). **Sakura Kirino:** Resources (equal). **Koji Yamashita:** Resources (equal). **Yuka Hayakawa:** Resources (equal). **Leona Osawa:** Resources (equal). **Mayu Higuchi:** Resources (equal). **Kenta Takaura:** Resources (equal). **Chiaki Maeyashiki:** Resources (equal). **Shun Kaneko:** Resources (equal). **Nobuharu Tamaki:** Resources (equal). **Hiroyuki Nakanishi:** Resources (equal). **Jun Itakura:** Resources (equal). **Yuka Takahashi:** Resources (equal). **Yasuhiro Asahina:** Supervision (equal). **Ryuichi Okamoto:** Supervision (equal). **Masayuki Kurosaki:** Funding acquisition (equal); supervision (equal); validation (equal); writing – review and editing (equal).

## CONFLICT OF INTEREST

Kaoru Tsuchiya, Masayuki Kurosaki, and Namiki Izumi received advisory board fees and honoraria for speakers' bureau from Bayer, Eli Lilly Japan, Chugai Pharmaceutical Company, and Eisai. The Japanese Ministry of Health, Labour and Welfare had no role in the design of the study; in the collection, analysis, or interpretation of data; in the writing of the manuscript, or in the decision to publish the results.

## INFORMED CONSENT STATEMENT

Informed consent was obtained from all subjects involved in the study.

## ETHICS STATEMENT

The study was conducted according to the guide‐lines of the Declaration of Helsinki and approved by the Institutional Review Board (or Ethics Committee) of Musashino Red Cross Hospital (protocol code: 1099, June 17, 2020).

## Supporting information


**Table S1** Baseline sorafenib patient characteristics
Table S2. Baseline lenvatinib patient characteristics
Table S3. Factors associated with PFS

**Figure S1** Overall survival and progression‐free survival outcomes in the high mG8 score group. (A) Kaplan–Meier estimates of overall survival by TKI. (B) Kaplan–Meier estimates of progression‐free survival by TKI.
Figure S2. Overall survival and progression‐free survival outcomes in the low mG8 score group. (A) Kaplan–Meier estimates of overall survival by TKI. (B) Kaplan–Meier estimates of progression‐free survival by TKI.

## Data Availability

Data Availability Statment: The data presented in this study are available upon request from the corresponding author.
