# Supplementary material for: Clinical usefulness of geriatric assessment in elderly patients with unresectable hepatocellular carcinoma receiving sorafenib or lenvatinib therapy
Source: Cancer Rep (Hoboken). 2022 Mar 18;5(11):e1613. doi: 10.1002/cnr2.1613 (PMC9675392; doi:10.1002/cnr2.1613)
Supplement: Supplementary file 1 — Table S1 Baseline sorafenib patient characteristics Table S2. Baseline lenvatinib patient characteristics Table S3. Factors associated with PFS [file CNR2-5-e1613-s001.docx]

**Table S1. Baseline sorafenib patient characteristics**

| Factor | Group | mG8 ≤10 (n = 22) | mG8 ≥11 (n = 43) | p-value |
| --- | --- | --- | --- | --- |
| BCLC stage, n(%) | A | 0 ( 0.0) | 1 ( 2.3) | 0.863 |
|  | B | 10 (45.5) | 22 (51.2) |  |
|  | C | 12 (54.5) | 20 (46.5) |  |
| ECOG-PS, n(%) | 0 | 14 (63.6) | 30 (69.8) | 0.895 |
|  | 1 | 7 (31.8) | 11 (25.6) |  |
|  | 2 | 1 ( 4.5) | 2 ( 4.7) |  |
| Gender, n(%) | F | 4 (18.2) | 16 (37.2) | 0.159 |
|  | M | 18 (81.8) | 27 (62.8) |  |
| Age, years median(range) |  | 80 (76, 98) | 80 (75, 89) | 0.781 |
| Etiology, n(%) | ALD | 4 (18.2) | 4 ( 9.3) | 0.198 |
|  | HBV | 2 ( 9.1) | 1 ( 2.3) |  |
|  | HBV+HCV | 1 ( 4.5) | 0 ( 0.0) |  |
|  | HCV | 12 (54.5) | 26 (60.5) |  |
|  | Others | 3 (13.6) | 12 (27.9) |  |
| Major vascular invasion (%) | No | 20 (90.9) | 36 (83.7) | 0.706 |
|  | Yes | 2 ( 9.1) | 7 (16.3) |  |
| Extrahepatic spread (%) | Yes | 11 (50.0) | 16 (37.2) | 0.426 |
|  | No | 11 (50.0) | 27 (62.8) |  |
| Tumor volume≥50% (%) | No | 17 (81.0) | 40 (93.0) | 0.204 |
|  | Yes | 4 (19.0) | 3 ( 7.0) |  |
| ALBI score |  | −2.07 (−2.87, −1.34) | −2.17 (−2.93, −1.33) | 0.546 |
| Baseline AFP median(range) |  | 37.8 (2.00, 27900) | 33.2 (1.80, 69100) | 0.484 |
| Baseline DCP, median(range) |  | 512 (10.0, 54104) | 244 (8.90, 26499) | 0.534 |

BCLS stage: Barcelona Clinic Liver Cancer stage; ECOG-PS: the Eastern Cooperative Oncology Group Scale of Performance Status; ALBI score:albumin–bilirubin score; DCP: des-gamma-carboxy prothrombin; AFP: α-fetoprotein;

**Table S2. Baseline lenvatinib patient characteristics**

| Factor | Group | mG8 ≤10 (n = 10) | mG8 ≥11 (n = 26) | p-value |
| --- | --- | --- | --- | --- |
| BCLC stage, n(%) | B | 7 (70.0) | 17 (65.4) | 1 |
|  | C | 3 (30.0) | 9 (34.6) |  |
| ECOG-PS, n(%) | 0 | 7 (70.0) | 14 (53.8) | 0.468 |
|  | 1 | 3 (30.0) | 12 (46.2) |  |
| Gender, n(%) | F | 2 (20.0) | 7 (26.9) | 1 |
|  | M | 8 (80.0) | 19 (73.1) |  |
| Age, years median(range) |  | 81 (76, 85) | 83 (75, 93) | 0.304 |
| Etiology, n(%) | ALD | 3 (30.0) | 5 (19.2) | 0.788 |
|  | HBV | 0 (0.0) | 1 (3.8) |  |
|  | HCV | 5 (50.0) | 11 (42.3) |  |
|  | Others | 1 (11.1) | 10 (37.0) |  |
| Major vascular invasion (%) | No | 8 (80.0) | 24 (92.3) | 0.305 |
|  | Yes | 2 (20.0) | 2 (7.7) |  |
| Extrahepatic spread (%) | Yes | 2 (20.0) | 5 (19.2) | 1 |
|  | No | 8 (80.0) | 21 (80.8) |  |
| Tumor volume≥50% (%) | No | 8 (80.0) | 25 (96.2) | 1 |
|  | Yes | 2 (20.0) | 1 (3.8) |  |
| ALBI score |  | −1.93 (−3.21, −1.35) | −2.40 (−2.87, −1.79) | 0.051 |
| Baseline AFP median(range) |  | 116.4 (7.50, 3270) | 9.10 (1.60, 97455) | 0.144 |
| Baseline DCP, median(range) |  | 1940 (13.9, 427126) | 543 (10.2, 24874) | 0.205 |

BCLS stage: Barcelona Clinic Liver Cancer stage; ECOG-PS: the Eastern Cooperative Oncology Group Scale of Performance Status; ALBI score: albumin–bilirubin score; DCP: des-gamma-carboxy prothrombin; AFP: α-fetoprotein;

**Table S3. Factors associated with PFS**

|  | All patients | | | | sorafenib | | | | lenvatinib | | | |
| --- | --- | --- | --- | --- | --- | --- | --- | --- | --- | --- | --- | --- |
|  | Univariate |  | Multivariate |  | Univariate |  | Multivariate |  | Univariate |  | Multivariate |  |
| Factor | HR(95% CI) | p-value | HR(95% CI) | p-value | HR(95% CI) | p-value | HR(95% CI) | p-value | HR(95% CI) | p-value | HR(95% CI) | p-value |
| **Gender** |  |  |  |  |  |  |  |  |  |  |  |  |
| Female | 1 |  |  |  | 1 |  |  |  | 1 |  |  |  |
| Male | 0.85(0.50-1.42) | 0.52 |  |  | 1.00(0.54-1.86) | 0.99 |  |  | 0.54(0.20-1.42) | 0.21 |  |  |
| **Age** |  |  |  |  |  |  |  |  |  |  |  |  |
| 75–79 | 1 |  |  |  | 1 |  |  |  | 1 |  |  |  |
| ≥80 | 0.95(0.59-1.53) | 0.83 |  |  | 0.97(0.55-1.70) | 0.92 |  |  | 1.05(0.40-2.70) | 0.93 |  |  |
| **ECOG-PS** |  |  |  |  |  |  |  |  |  |  |  |  |
| 1/2 | 1 |  |  |  | 1 |  |  |  | 1 |  |  |  |
| 0 | 1.23(0.75-2.02) | 0.4 |  |  | 0.90(0.50-1.64) | 0.74 |  |  | 1.73(0.71-4.22) | 0.23 |  |  |
| **Major vascular invasion** |  |  |  |  |  |  |  |  |  |  |  |  |
| VP0/1/2 | 1 |  |  |  | 1 |  |  |  | 1 |  |  |  |
| VP3/4 | 0.81(0.35-1.88) | 0.63 |  |  | 0.74(0.29-1.86) | 0.52 |  |  | 0.93(0.12-7.27) | 0.94 |  |  |
| **Extrahepatic spread** |  |  |  |  |  |  |  |  |  |  |  |  |
| Yes | 1 |  |  |  | 1 |  |  |  | 1 |  |  |  |
| No | 1.06(0.64-1.74) | 0.82 |  |  | 0.97(0.55-1.69) | 0.91 |  |  | 2.25(0.64-7.89) | 0.21 |  |  |
| **Modified ALBI grade** |  |  |  |  |  |  |  |  |  |  |  |  |
| 2b/3 | 1 |  | 1 |  | 1 |  |  |  | 1 |  |  |  |
| 1/2a | 0.50(0.31-0.80) | 0.0039 | 0.57(0.35-0.94) | 0.027 | 0.48(0.27-0.88) | 0.017 |  |  | 0.72(0.29-1.80) | 0.48 |  |  |
| **mG8 score** |  |  |  |  |  |  |  |  |  |  |  |  |
| ≤10 | 1 |  |  |  | 1 |  |  |  | 1 |  |  |  |
| ≥11 | 0.84(0.51-1.41) | 0.52 |  |  | 0.73(0.41-1.31) | 0.29 |  |  | 0.84(0.32-2.21) | 0.73 |  |  |
| I**ntrahepatic tumor volume** |  |  |  |  |  |  |  |  |  |  |  |  |
| <50% | 1 |  |  |  | 1 |  |  |  | 1 |  | 1 |  |
| ≥50% | 1.68(0.76-3.68) | 0.2 |  |  | 1.03(0.41-2.60) | 0.95 |  |  | 44.1(3.77-515) | 0.0025 | 24.8(2.04-301) | 0.012 |
| **DCP** |  |  |  |  |  |  |  |  |  |  |  |  |
| <400 mAU/mL | 1 |  |  |  | 1 |  |  |  | 1 |  |  |  |
| ≥400 mAU/mL | 1.19(0.74-1.89) | 0.48 |  |  | 1.39(0.80-2.42) | 0.24 |  |  | 1.24(0.48-3.25) | 0.66 |  |  |
| **AFP** |  |  |  |  |  |  |  |  |  |  |  |  |
| <400 mAU/mL | 1 |  | 1 |  | 1 |  |  |  | 1 |  | 1 |  |
| ≥400 mAU/mL | 1.78(1.07-2.97) | 0.028 | 1.56(0.93-2.64) | 0.095 | 1.38(0.74-2.57) | 0.32 |  |  | 2.95(1.16-7.51) | 0.023 | 2.39(0.88-6.46) | 0.087 |
| **TKI** |  |  |  |  |  |  |  |  |  |  |  |  |
| Sorafenib | 1 |  | 1 |  |  |  |  |  |  |  |  |  |
| Lenvatinib | 0.57(0.34-0.95) | 0.031 | 0.63(0.37-1.07) | 0.09 |  |  |  |  |  |  |  |  |

ECOG-PS: the Eastern Cooperative Oncology Group Scale of Performance Status; modified ALBI score: modified albumin–bilirubin score; mG8 score: modified Geriatric 8 score ,DCP: des-gamma-carboxy prothrombin; AFP: α-fetoprotein; TKI: tyrosine kinase inhibitor
