# Supplementary material for: Clinical usefulness of geriatric assessment in elderly patients with unresectable hepatocellular carcinoma receiving sorafenib or lenvatinib therapy
Source: Cancer Rep (Hoboken). 2022 Mar 18;5(11):e1613. doi: 10.1002/cnr2.1613 (PMC9675392; doi:10.1002/cnr2.1613)
Supplement: Supplementary file 2 — Figure S1 Overall survival and progression‐free survival outcomes in the high mG8 score group. (A) Kaplan–Meier estimates of overall survival by TKI. (B) Kaplan–Meier estimates of progression‐free survival by TKI. Figure S2. Overall survival and progression‐free survival outcomes in the low mG8 score group. (A) Kaplan–Meier estimates of overall survival by TKI. (B) Kaplan–Meier estimates of progression‐free survival by TKI. [file CNR2-5-e1613-s002.docx]

***
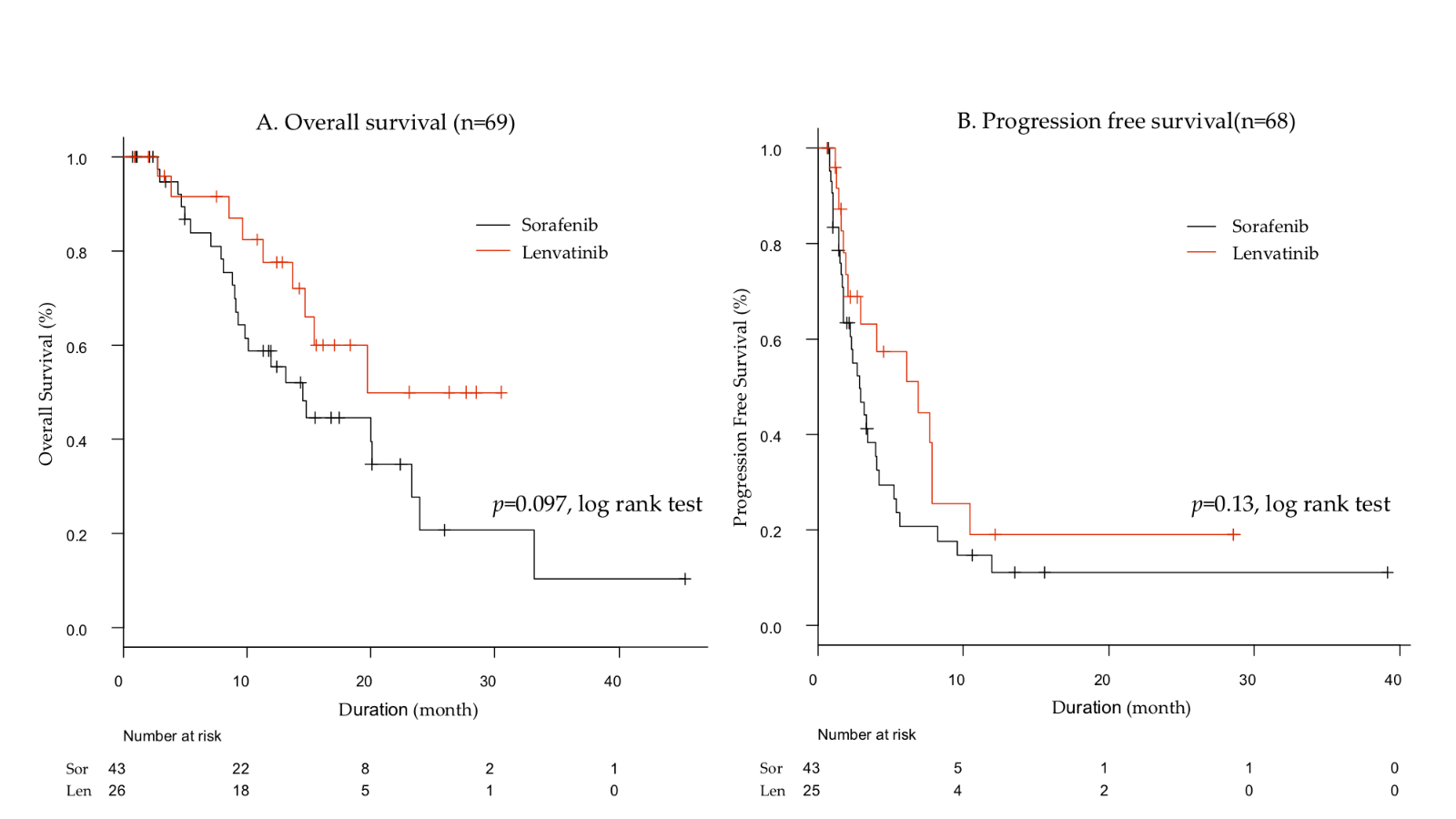
***

**Figure S1. Overall survival and progression-free survival outcomes in the high mG8 score group**. (A) Kaplan–Meier estimates of overall survival by TKI. (B) Kaplan–Meier estimates of progression-free survival by TKI.


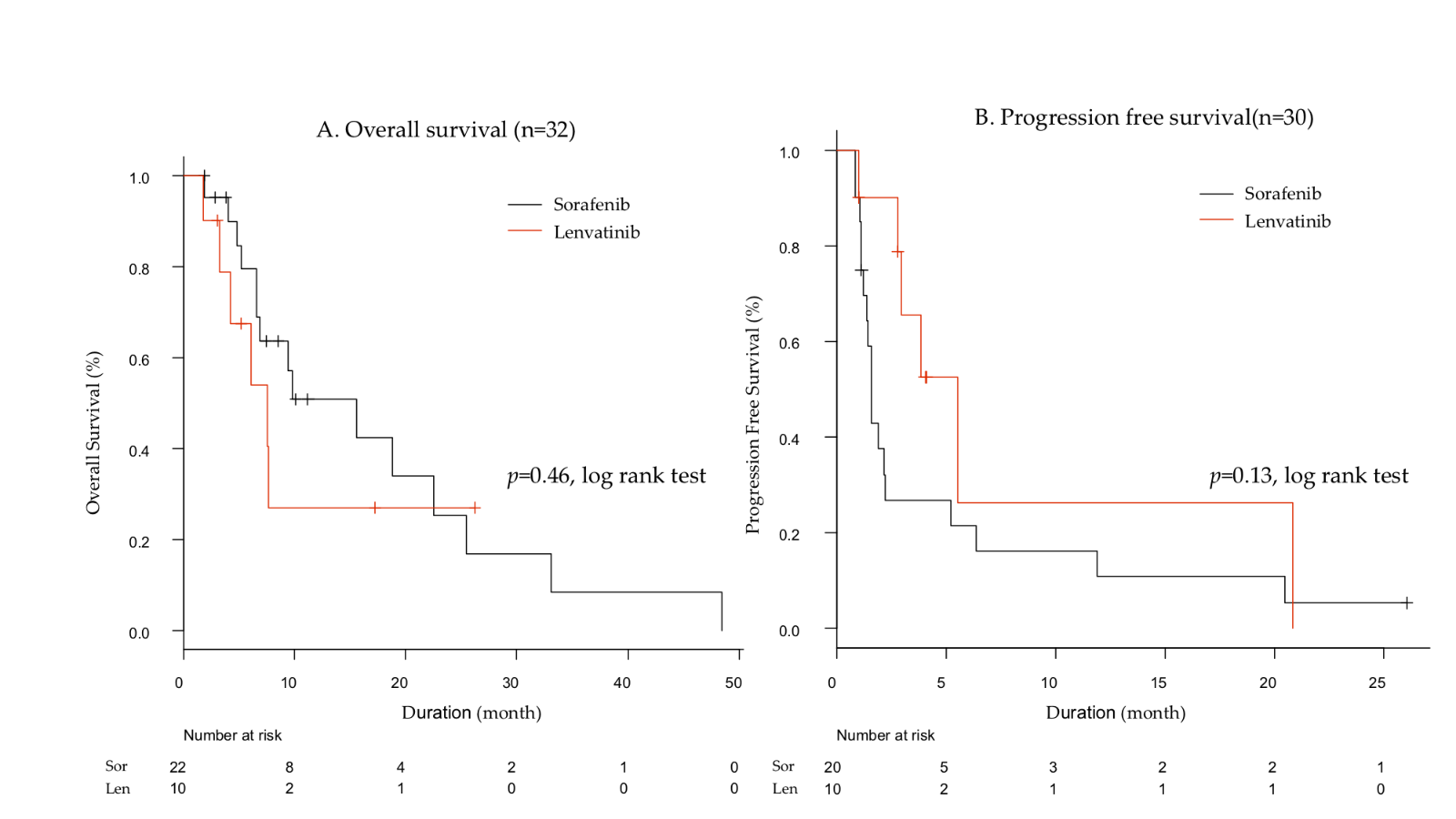


**Figure S2. Overall survival and progression-free survival outcomes in the low mG8 score group**. (A) Kaplan–Meier estimates of overall survival by TKI. (B) Kaplan–Meier estimates of progression-free survival by TKI.
